# Untargeted Metabolomics to Investigate the Influence of Epigenetic Modifiers on the Metabolism of *Fusarium verticillioides*

**DOI:** 10.1155/2024/1763495

**Published:** 2024-10-28

**Authors:** E. Groppi, A. Gadea, C. Monge, V. Cristofoli, M. Vansteelandt, M. Haddad

**Affiliations:** UMR 152 Pharma CDev, Université de Toulouse, IRD, UPS, Toulouse, France

## Abstract

Toxigenic fungi are capable of producing toxic metabolites, called mycotoxins. But the presence of silent and lowly expressed genes represents the main challenge for the discovery of novel mycotoxins, especially their lesser-known forms, commonly referred to as “emerging mycotoxins.” Epigenetic modifiers (EMs) are compounds that are able to alter the production of metabolites through the induction of silent biosynthetic pathways leading to an enhanced chemical diversity. The aim of this study was to assess the effects of different chemical modulators on the metabolic profiles of the well-known toxigenic fungal species, *Fusarium verticillioides*. Four EMs, 5-azacytidine, sodium butyrate, nicotinamide (NIC), and sodium valproate (SV), were used. Following their addition to *Fusarium verticillioides* cultures, the metabolic profiles were analyzed by using UHPLC–HRMS/MS under targeted and untargeted metabolomics approaches. Metabolites were putatively annotated through the use of MS-DIAL and MS-FINDER. Our results show that the treatment with SV induced the most important alteration of the secondary metabolic profile of *F. verticillioides*, by promoting the expression of cryptic genes. Among the 50 most discriminating metabolites across five culture conditions, 12 were fusarins or fusarin analogs. In contrast, SB and NIC had little impact on these metabolites. The study highlights SV's ability to alter gene expression by inhibiting DNA deacetylation in fungal strains. This research could have significant implications for agriculture and food industry, especially in regions facing major mycotoxin challenges.

## 1. Introduction

Filamentous fungi are microorganisms capable of producing compounds of pharmaceutical and commercial interest. Their metabolites include important pharmaceuticals such as penicillin or statins, potent poisons such as aflatoxins or fumonisins, and metabolites that are both toxic and pharmaceutically useful, such as ergot alkaloids [1–3]. These toxin-producing fungi can grow on nearly any organic material and, as a result, food and feed are regularly contaminated with metabolites produced by these fungal strains. Toxic fungal metabolites, commonly referred to as mycotoxins, are of major importance to food and feed safety worldwide and represent a public health problem. The term “mycotoxins” refers to “natural products produced by fungi that evoke a toxic response when introduced in a low concentration to higher vertebrates and other animals by a natural route” [4]. Various fungi are known to produce mycotoxins, including *Aspergillus*, *Fusarium,* and *Penicillium* species. The most important classes of mycotoxins include the highly carcinogenic aflatoxins (e.g., aflatoxin B1 [AFB1]), trichothecenes (e.g., deoxynivalenol [DON]), fumonisins (e.g., fumonisin B1 [FB1]), ochratoxin A (OTA), and zearalenone (ZEN), for which maximum levels in food and feed are enforced and regulated in Europe. But in addition to these well-known mycotoxins, much attention has been given to less well-described mycotoxins, known as “emerging” mycotoxins, because they are poorly characterized or recently identified and, therefore, are currently unregulated. The most frequently emerging mycotoxins are produced by the most common grain-contaminating fungi, *Fusarium* spp., and include enniatins, beauvericin (BEA), apicidin, aurofusarin, culmorin, butenolide, fusaric acid, moniliformin (MON), and fusaproliferin [5–13].

The specific conditions leading to gene expression and, subsequently, to the production of all metabolites in fungi are not fully understood. Many biosynthetic gene clusters, often found in the distal telomeric regions of chromosomes [14–16], are controlled by epigenetic mechanisms like histone acetylation and methylation [17–19]. These clusters can remain silent or weakly expressed, limiting the discovery of secondary metabolites. Environmental factors that trigger metabolite production in nature may not be replicated in lab conditions [20], leading to only partial expression of a fungus's metabolic potential [21, 22]. Epigenetic regulation, including histone acetylation by histone acetyl-transferases (HATs) and deacetylation by histone deacetylases (HDACs), influences chromatin structure and gene accessibility. DNA methylation, which adds a methyl group to cytosine via DNA methyltransferases (DNMT) [23, 24], further controls gene expression [25]. Silent metabolic pathways, regulated by these epigenetic factors, can be activated by methods such as the addition of epigenetic modifiers (EMs), unlocking new metabolite production [22, 26, 27].

EMs can lead to chromatin remodeling and the activation of certain biosynthetic genes [16, 19, 24, 28], and those used in this experiment were nicotinamide (NIC), sodium valproate (SV), sodium butyrate (SB), and 5-azacytidine (AZA). SV, SB, and NIC are HDAC inhibitors [29], which keep DNA in the euchromatin state, making it accessible for transcription. AZA, as a DNMT inhibitor, prevents DNA methylation, also making it accessible for transcription [16, 20].

Our research group is currently focusing on the issue of mycotoxins in Africa, and we have selected the *Fusarium verticillioides* strain as our model organism to assess the effects of different chemical modulators on the metabolic profiles of this toxigenic fungal strain. The metabolic profiles were analyzed by using ultra-high-performance liquid chromatography–MS analysis (UHPLC–HRMS/MS) under targeted and untargeted metabolomics approaches. This research investigates how EMs affect the metabolome of *Fusarium verticillioides*, particularly mycotoxin production. The study hypothesizes that EMs can significantly alter *F. verticillioides*' metabolic profile and mycotoxin biosynthesis. Using an integrative metabolomics approach with LC-MS and bioinformatics, the project will analyze the effects of various EMs on the fungus. While gene expression analysis could provide valuable insights, our focus on metabolomics allows us to directly observe changes in the fungal metabolome, including both known and potentially novel mycotoxins. This approach provides a comprehensive view of the end products of gene expression and cellular processes, offering a different but complementary perspective to gene expression studies.

The expected outcomes aim to enhance the understanding of epigenetic regulation of mycotoxin production, potentially leading to new strategies for controlling mycotoxin contamination and improving food safety. This research could have significant implications for agriculture and food industry, especially in regions facing major mycotoxin challenges.

## 2. Materials and Methods

### 2.1. Chemicals and Solvents

All solvents used for chromatography were of HPLC grade. A Milli-Q RG system (Millipore, France) was used to produce high-purity water with a resistivity of 18.2 MΩ·cm. The solvents used for the experiments are as follows: formic acid (FA) (> 98%, Sigma-Aldrich, St. Louis, MO, USA), methanol (MeOH, Fischer chemical), ethyl acetate (EtOAc, Fischer chemical), and acetonitrile (ACN, Fischer chemical). Mycotoxin standards (AFB1, OTA, and patulin [PAT]) were purchased from Sigma-Aldrich (St. Louis, MO, USA) and immediately stored at −20°C. Standards of nivalenol (NIV), DON, 3-AcDON, 15-acetyldeoxynivalenol (15-AcDON), diacetoxyscirpenol (DAS), fusarenon X (FUS-X), the mixture of T-2 toxin (T-2) and HT-2 toxin (HT-2), the mixture of FB1 and fumonisin B2 (FB2), the mixture of enniatins (A, A1, B, and B1) and BEA, ZEN, and MON were purchased from Libios (Vindry-sur-Turdine, France). The EMs used are as follows: NIC (Sigma), SB (Sigma), AZA (Sigma), and SV (Sigma).

### 2.2. Fungal Material

The *F. verticillioides* INRA63 strain was isolated and identified by the MycSA group at INRAE, Bordeaux, Nouvelle-Aquitaine, France [30]. The strain was received in cryotubes containing 30% glycerol. Thawed fragments were grown on Petri dishes containing agar malt extract (MEA) to prepare inocula.

### 2.3. Fermentation Conditions

*Fusarium verticillioides* was grown on PDA over 3 days prior to inoculation of the seed culture containing 150 mL of potato dextrose broth (PDB), incubated at 27°C. After 3 days, 250 mL Erlenmeyer flasks containing 150 mL of PDB were inoculated with 1 mL of the seed culture and treated with the corresponding epigenetic modulators (SB, NIC, AZA, and SV) dissolved in DMSO. NIC and SB [31] were added at 1 μM, AZA [32, 33] was added at 25 μM, and SV [34] was added at 100 μM as final concentrations. Fermentations were carried out over 2 weeks at 27°C under static conditions. For all the EMs, the percentage of DMSO used for dissolution was equal or lower than 0.1% of the total PDB medium. All conditions were done in six replicates. DMSO was added to control cultures without EMs. Control media with and without EMs were also included in triplicate.

### 2.4. Extraction of Culture

After 14 days of incubation, the entire culture broth (including mycelium) was extracted with 150 mL of EtOAc (v:v 1/1) after 1 h of ultrasonic bath treatment. The mycelium was filtered from the culture medium by a glass wool filtration step. The culture broth was left to settle for a few minutes in a separatory funnel. The organic phase was filtered through Whatman paper and dehydrated by adding MgSO_4_. The filtrate was collected in a flask and dried using a rotary evaporator (BÜCHI Rotavapor R-114). Once dry, the extracts were resuspended in MeOH in pre-weighed 2 mL Eppendorf tubes. Methanolic solutions at 2 mg/mL of all extracts were prepared for UHPLC–HRMS. Quality control (QC) samples were prepared by pooling an aliquot (20 μL) of all extracts for each experiment (EMs).

### 2.5. UHPLC–HRMS Profiling

Analyses were performed on a UHPLC Ultimate 3000 system (Dionex) coupled with an LTQ Orbitrap XL mass spectrometer (Thermo Fisher Scientific, Hemel Hempstead, UK). Samples were separated on an HPLC Zorbax Eclipse XDB-C18 column, 2.1 × 150 mm, 3.5 μm (Agilent Technologies, Santa Clara, CA, USA). The mobile phase A was ultrapure water acidified with 0.1% FA and mobile phase B was ACN acidified with 0.1% FA. The solvent gradient was as follows: 0 min, 95% A; 10 min, 95% B; 12.5 min, 95% B; 13 min, 95% A; 15 min, 95% A. The flow rate was 0.3 mL/min, the column temperature was set to 40°C, the autosampler temperature was set to 15°C, and injection volume was fixed to 5 μL. Mass detection was performed using an electrospray source (ESI) in positive ionization (PI) mode at 15,000 resolving power (full width at half maximum [FWHM] at 400 m/z). The mass scanning range was m/z 100–2000 for all samples. Ionization spray voltage was set to 3.5 kV, and the capillary temperature was set to 300°C. Each full MS scan was followed by data-dependent acquisition of MS/MS spectra for the three most-intense ions using stepped collision-induced dissociation (CID) at 35 arbitrary energy units.

### 2.6. Data Processing

LC-MS data were processed in the same way as previous metabolomics studies conducted in our team [35, 36] using the MS-DIAL/MS-FINDER/MS-CleanR workflow [37]. Data obtained from PI were processed with MS-DIAL Version 4.90 [38]. MS1 and MS2 tolerances were set to 0.01 and 0.05 Da, respectively, in centroid mode. Data were collected between 0 and 15 min. Data were collected between 100 and 1500 Da. The minimum peak height was set based on the baseline of the total ion chromatogram (TIC) observed for the blank. Peaks were aligned to a reference QC with a retention time tolerance of 0.1 min and an MS1 tolerance of 0.025 Da. MS-DIAL data were cleaned with MS-CleanR using default parameters, except for the maximum relative standard deviation (RSD) which was set to 40: all filters were checked; the minimum blank ratio was set to 0.8 and the relative mass defect (RMD) was between 50 and 3000. The maximum mass difference was set to 0.005 Da and the maximum Rt difference to 0.025 min. Pearson correlation was used to calculate clusters with a minimum correlation set to 0.8 and *α* = 0.05. Two peaks were retained per cluster, the most intense and the most connected to other ions. The data retrieved at the output of the workflow were then annotated with MS-FINDER Version 3.52 [39]. MS1 and MS2 tolerances were set to 5 and 15 ppm, respectively, and O, N, and Cl atoms were selected in the formula search tool. Annotation was performed by comparing with an .MSP database of mycotoxin standards (including DON, AFB1, 3-AcDON, DAS, FUS-X, NIV, OTA, ZEN, HT-2, T-2, enniatins [B, B1, A, and A1], FB1, FB2, PAT, and BEA) (annotation level 1). *In silico* matches (annotation level 2) were performed using internal databases from the literature: 2.1. F. verticillioides metabolites, 2.2. Fusarium metabolites, 2.3. Mycotoxins, and 2.4. Generic MS-FINDER databases (ChEBI, NPA, NANPDB, COCONUT, KNApSAcK, PubChem, UNPD, and T3DB). Data were exported as .CSV files for metadata information (Rt, m/z, annotation results, peak areas, etc.). Only annotations related to mycotoxins were retained and manually verified by comparing high-resolution mass and MS/MS fragmentation to literature data.

### 2.7. Statistical Analyses

For multivariate analyses, MS-Finder .CSV files were uploaded to the online platform MetaboAnalyst Version 5 [40]. Peaks were filtered based on QCs if their RSDs were greater than 20%. A statistical filter based on the interquartile range (IQR) was applied, filtering a certain percentage depending on the number of variables. Samples were first normalized by sum, and variables were weighted by Pareto scaling (mean-centered and divided by the square root of the standard deviation of each variable). Principal component analysis (PCA) was performed to show the distribution of samples into groups. For better visualization, partial least squares discriminant analysis (PLS-DA) was performed to show the impact of incubation time on the metabolome of *F. verticillioides*. Variable importance in projection (VIP) scores of the most discriminating molecules were presented. The VIP score is a measure used in PLS regression analysis to assess the importance of each variable in the model projection. A VIP score > 1 indicates that the variable is important for the model, while a score < 0.5 suggests that it has little importance [41]. The *m/z_*Rt pair of the top 50 features with significant changes, associated to their normalized peak area, was plotted on a heat map (hierarchical clustering) using the analysis of variance (one-way ANOVA) and *T*-test. Only group averages were showed, and clusters were not clustered to show the natural contrast among groups. Volcano plot representation was used to see the influence of EMs in details, and it combines fold-change (FC) analysis and *T*-test results in one graphical figure to visualize significant features. On the *X*-axis, FC threshold was set to 2, and we compared the addition of EM condition with the control condition (FV + PDB) while on the *Y*-axis, the *p* value threshold was set at 0.05.

### 2.8. Molecular Network and t-SNE Visualization

The .MGF file from the MS-DIAL/MS-FINDER/MS-CleanR workflow and the quantification table in PI mode were imported into MetGem software Version 1.4.3 [42, 43]. The molecular network (MN) was then customized in Cytoscape software Version 3.8.2. To establish the MN, the cosine score was calculated. This is a spectral similarity score in MS^2^, and the closer it is to 1, the more structurally similar the compared molecules are. The m/z tolerance was set to 0.2 Da, and spectra sharing at least 4 peaks were retained. MS2 spectra were filtered by keeping peaks above 50 Th. All peaks located within the range of ± 17 Da around the precursor m/z were removed. The MN was created with a cosine score greater than 0.7. Links between two nodes were retained if each node was in the top 10 most similar nodes of the other. The width of the links depends on the cosine score value between two related molecules. Nodes representing mycotoxin standards are represented with a rounded rectangle label, while other nodes have a round label. Nodes are filled with a pie chart representing the average normalized peak area of each molecule detected in the different culture media. For t-stochastic neighbor embedding (t-SNE) network visualization, nodes were retained if there was a cosine score greater than 0.7 between two molecules. The number of iterations, perplexity, learning rate, and early exaggeration parameters were set to 1000, 6, 200, and 12, respectively. Barnes–Hut approximation was enabled using an angle of 0.5.

## 3. Results and Discussion

### 3.1. In Vitro Growth Assays and Chemical Characteristics of Fungal Cultures

**Figure 1:**
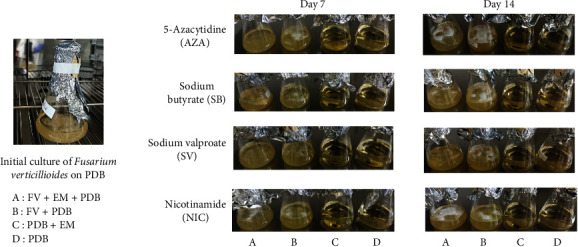
Morphological appearance of the initial *F. verticillioides* culture in PDB and in the presence of the various EMs after 7 and 14 days of culture.

**Figure 2:**
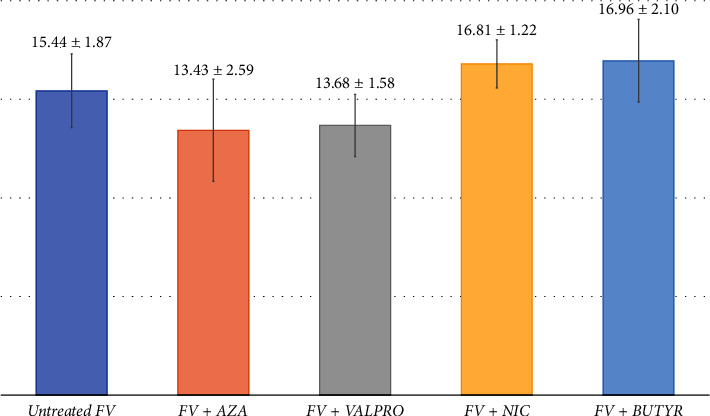
Total extract weights after EM treatment.

*F. verticillioides* was cultured in PDB medium under various conditions: control (without additives) and in the presence of EMs—NIC, SB, SV, or AZA—for 14 days. For the chemical elicitation experiment, *F. verticillioides* was first grown in PDB for 3 days and then transferred to PDB containing one of the four EMs. Six replicates were performed for each condition, with three additional controls: PDB alone, PDB + EM, and PDB + *F. verticillioides* without EM. The cultures initially exhibited white mycelia, later developing red and orange pigments across all conditions. No significant morphological differences were observed between the EM-treated cultures and the control group. The presence of EMs did not noticeably affect the fungal growth rate or colony morphology.  Figure 1 illustrates the morphological aspects of *F. verticillioides* in various culture conditions over time. Furthermore, there were no significant differences in extract weights among the different groups (Figure 2).

**Figure 3:**
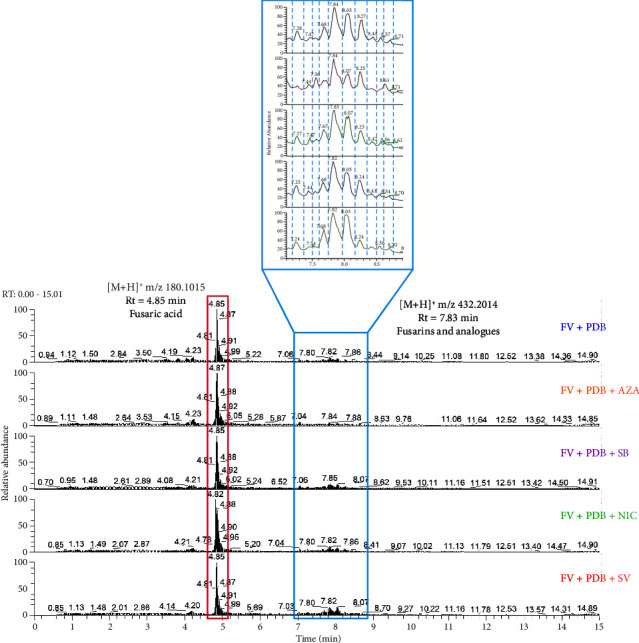
UHPLC-(+) ESI–HRMS chromatograms of crude extracts of *F. verticillioides* grown in PDB medium alone (FV + PDB) or treated with 5-azacytidine (FV + PDB + AZA), sodium butyrate (FV + PDB + SB), nicotinamide (FV + PDB + NIC), or sodium valproate (FV + PDB + SV).

The general chemical profiles of *F. verticillioides* grown with and without EMs appeared similar (Figure 3). A major peak at m/z 180.1011 and Rt = 4.85 min was observed, along with a cluster of peaks between 7 and 9 min, including open-chain fusarin C (m/z 432.2014, Rt = 7.83 min). Closer examination revealed the modulation of peak intensities corresponding to different fusarins. The peak at m/z 180.1011 was initially annotated in MetGem-associated databases as an analog of kynurenic acid and anthranilic acid, metabolites derived from tryptophan degradation. This pathway is known to be used by some *Fusarium* sp. for producing toxic compounds like BEA or fusaric acid. However, manual verification of the MS^2^ spectrum identified this peak as fusaric acid.

### 3.2. Untargeted Study of Metabolites Produced by *F. verticillioides* in the Presence of EMs

Extracts from *F. verticillioides* cultured with or without EMs (AZA, SB, SV, or NIC) were analyzed by using UHPLC–HRMS/MS. The raw data from the LC-MS profiles of the extracts were processed in PI mode using MS-Dial/MS-CleanR/MS-Finder software according to an untargeted approach. The detection and annotation of mycotoxins and other metabolites present in the extracts were achieved by comparing the high-resolution mass (MS1), MS2 data, and Rt with mycotoxin standards and literature data when possible. We did not detect any fumonisins in our extracts, regardless of the conditions. However, we primarily annotated fusarins, fusarin analogs, fusaric acid, and bikaverin. Using MS-Finder software and the databases contained in MetGem, we annotated the metabolites presented in Table 1. Among the proposed annotations, a large number of compounds were identified as fusarin A or analogs (m/z 384 to m/z 434). To refine the annotation, we searched the literature for the MS^2^ spectra of the molecules to compare them with the experimental MS^2^ data.

**Figure 4:**
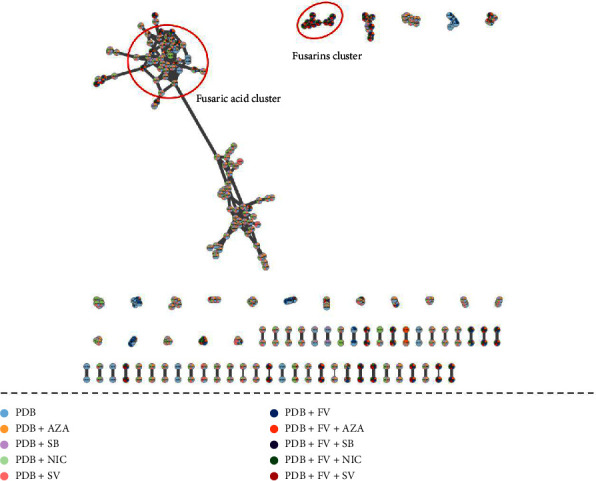
The molecular network of the extracts of *F. verticillioides* cultured in PDB alone or treated with one of the four epigenetic modifiers for 14 days. Each node corresponds to a single feature defined by the m/z_Rt pair (normalized area).

**Figure 5:**
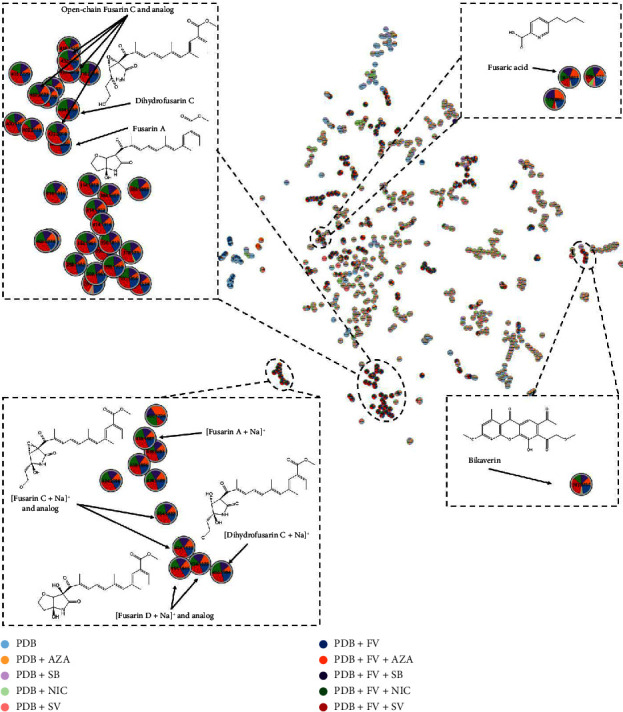
Visualization of the t-SNE network corresponding to the extracts of *F. verticillioides* cultured in PDB alone or treated with AZA, SB, NIC, or SV for 14 days. Each node corresponds to a single feature defined by the m/z_Rt pair (normalized area).

These data were then visualized on an MN based on the MS^2^ data, using MetGem and Cytoscape software (Figure 4). This visualization allowed us to better observe the relationships between the detected peaks, focusing on metabolites produced in the presence of EMs and not in the control condition, FV + PDB. The MN comprises 1155 nodes and 396 links, with 297 nodes connected and 858 not linked to any other molecule and are not visible on the MN (Figure 4). Many of the detected molecules are not specific to a condition, and some are only found in the control conditions, without FV. These are molecules present in the culture medium whose presence cannot be attributed to production by *F. verticillioides*. Therefore, we only focused on molecules detected in the presence of *F. verticillioides*. We highlighted the presence of a group of molecules to which fusaric acid belongs. This is the major peak present in all culture conditions, detected with the same intensity in all culture conditions with the four EMs. We also detected bikaverin, which is not linked to any other molecule and mainly present in the condition where *F. verticillioides* is cultured in the presence of SV. Then, it is found in the PDB + FV condition and then decreasingly in the PDB + FV + SB, PDB + FV + NIC and PDB + FV + AZA conditions. We found a cluster composed of some fusarins, but some others formed another cluster or were not linked to any other molecule. The molecules in this cluster are only detected in culture conditions with the fungus, so their presence is attributed to production by *F. verticillioides*. A clear trend appears in the distribution of these molecules: they are mostly detected with greater intensity in the condition where *F. verticillioides* is cultured in PDB with added SV and are generally weakly detected in the condition with AZA. To verify this trend for all fusarins and group them into a single cluster, we visualized these data as a t-SNE network (Figure 5).

The t-SNE visualization (Figure 5) allowed the clustering of fusarins, and we could distinguish two groups: one grouping fusarins in their [M+Na]^+^ form, and a second composed of fusarins in their [M+H]^+^ form. In the first cluster, we found fusarin A andopen-chain fusarin C, as well as some of its analogs at m/z 432.20. These two fusarins (A and C open-chain) are among the fusarins for which MS^2^ data on the [M+H]^+^ form are available in MetGem databases. MS^2^ data on other fusarins are not available for the [M+H]^+^ form, as this family of molecules is mainly detected as a sodium adduct, and most of the MS^2^ data available are related to the [M+Na]^+^ form. This explains why so many molecules in this cluster are annotated as analogs of fusarin A or open-chain fusarin C. The four molecules with m/z 432.20 share some common fragment ions with open-chain fusarin C. It could be fusarin C, epi-fusarin C, or fusarin D, as they all have the same molecular formula and exact mass [44]. For the molecule with m/z 434, annotated as dihydrofusarin C, we verified the annotation by comparing the MS^2^ spectrum with the MS^2^ spectrum of dihydrofusarin C available in the GNPS spectral library. However, the lack of MS^2^ data in the [M+H]^+^ form made the verification of annotations more complex. We noticed that fusarin family molecules in the [M+Na]^+^ form and those in the [M+H]^+^ form are found in different clusters. It would be interesting to cross-check the MS^2^ data of molecules in the [M+H]^+^ and [M+Na]+ forms to have a single annotation and a single node in the MN. In the group of fusarins in their [M+Na]^+^ form, we identified fusarin A, fusarin C, dihydrofusarin C, and fusarin D. The fusarins, whether found in the [M+Na]^+^ or [M+H]^+^ cluster, mostly have the same distribution in different conditions: predominantly in FV + PDB + SV, FV + PDB + SB, and FV + PDB, FV + PDB + NIC conditions, we find the fusarins in roughly equal proportions; however, they are less present in the FV + PDB + AZA condition.

In the group of bikaverin and its derivatives, we observed that bikaverin was mostly found when *F. verticillioides* was cultured in the presence of SV, then in the control condition, with SB, NIC, and less in the AZA condition. The derivatives of this metabolite are mostly found in the condition with SV addition, then with AZA addition, and finally in the control condition. They are produced in lesser quantities in the FV + PDB + NIC or FV + PDB + SB conditions. Fusaric acid is detected in all four conditions with the addition of an EM and in the control condition in equal proportions. In general, we observed that fusarins have a distribution that always follows the same trend: increased occurrence when *F. verticillioides* is cultured in PDB with SV and decreased occurrence when *F. verticillioides* is cultured in PDB with AZA compared to our control condition. The distribution of fusarins in the other two culture conditions, that is, with the addition of SB or NIC in the PDB medium, is quite similar to the control condition.

### 3.3. Study of the Influence of EMs on the Metabolism of *F. verticillioides*

**Figure 6:**
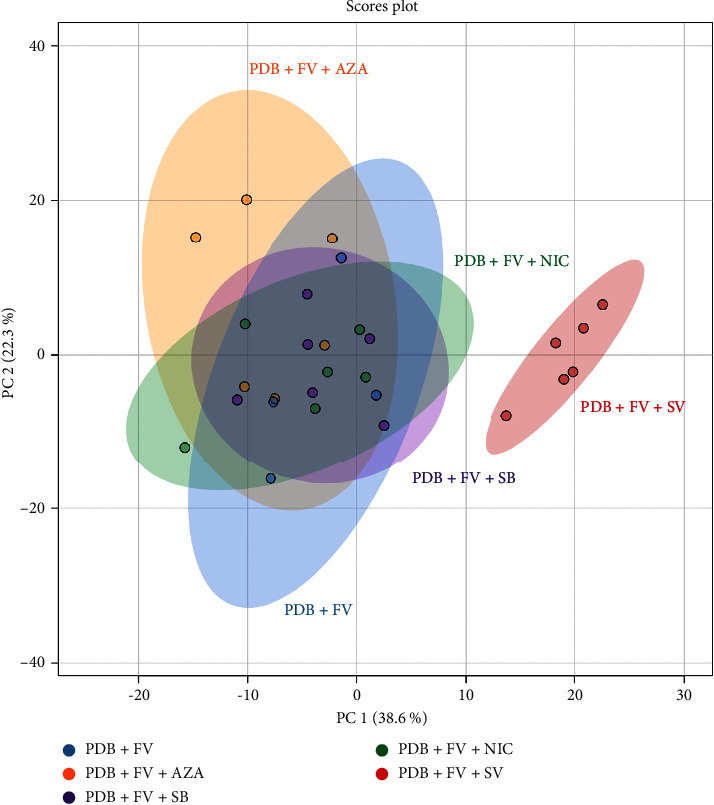
PCA corresponding to the extracts of *F. verticillioides* cultured in PDB alone or treated with AZA, SB, NIC, or SV after 14 days of incubation, based on the variables (normalized peak area as a function of m/z at an Rt) detected on the LC-MS chromatograms.

Multivariate analyses were then performed using the MetaboAnalyst 5.0 online platform. First, we conducted a PCA, which provided an overview of the impact of adding EMs to PDB-based culture media in which *F. verticillioides* inocula grew, on its metabolome. This is presented in Figure 6. This analysis grouped all independent biological replicates from the same condition. The PCA explains 60.9% of the total variance between groups. The first component (*x*-axis) explains 38.6% of the variability, and the second component (*y*-axis) explains 22.3% of the variability. This representation highlighted that the addition of SV changes the metabolome of *F. verticillioides* compared to our control condition and also compared to the addition of other EMs.

**Figure 7:**
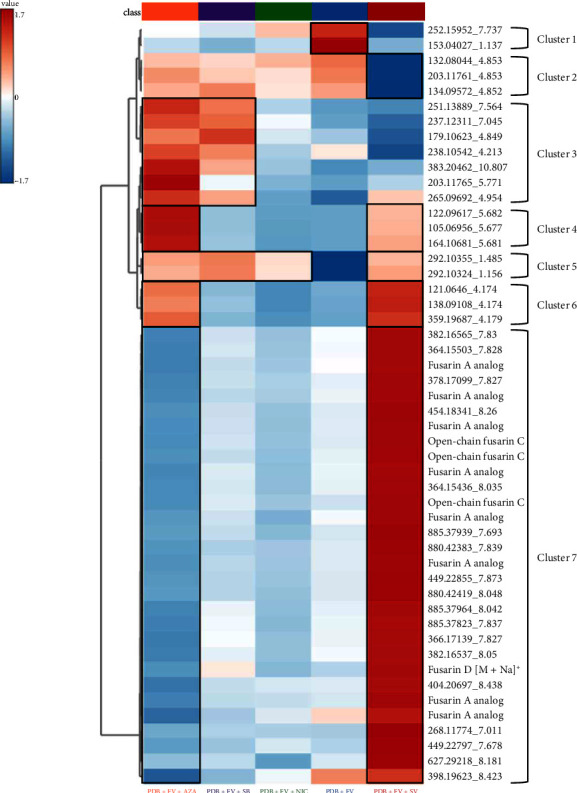
Heat map representing the hierarchical clustering of the most discriminating variables observed in extracts from *F. verticillioides* grown in PDB medium alone or treated with the various epigenetic modifiers (AZA, SB, NIC, and SV) obtained using ANOVA.

To observe the variables responsible for the differences between groups, we created a heat map (Figure 7). A one-way ANOVA test (*p* value < 0.05) was performed using the analysis of metabolite variance. Out of 579 variables, only 114 were significantly altered by the addition of different EMs in the culture medium. Figure 7 represents a heat map highlighting the occurrence of discriminant metabolites in the different culture conditions applied to *F. verticillioides*. The heat map allows hierarchical clustering of variables, providing a simplified visualization of data tables. Each row corresponds to the average of samples from the same condition (PDB + FV, PDB + FV + AZA, PDB + FV + SB, and PDB + FV + NIC, PDB + FV + SV). Each column represents a compound characterized by an m/z_Rt pair or its putative annotation. Each colored cell on the map corresponds to the mean concentration value of the molecule in the samples of the condition. This representation allows identifying variables that are overexpressed or underexpressed depending on the culture conditions applied. Therefore, we chose to represent the 50 most discriminant molecules between the groups. We found some of the molecules annotated in Table 1: analogs of fusarin A, open-chain fusarin C and its analogs, and fusarin D [M+Na]^+^. In total, of the 50 most discriminant metabolites between the 5 culture conditions, 12 are fusarins or fusarin analogs. We identified seven clusters with different metabolite distributions from our control condition: PDB + FV. The first cluster corresponds to molecules overexpressed in the control condition compared to other conditions (Cluster 1). The second cluster corresponds to molecules underexpressed by the addition of SV (Cluster 2). The third cluster corresponds to metabolites upregulated by the addition of AZA and SB (Cluster 3). The fourth cluster includes molecules overexpressed by the addition of AZA and SV (Cluster 4). The fifth cluster gathers metabolites overexpressed by the addition of each EM (Cluster 5). The sixth cluster corresponds to molecules overexpressed by the addition of AZA and SV (Cluster 6). Finally, the last cluster is composed of molecules overexpressed by the addition of SV and underexpressed by the addition of AZA (Cluster 7). Among the 12 molecules annotated as belonging to the fusarin family, all are overexpressed by the addition of SV and under-regulated by the addition of AZA (cluster 7). Thus, SV induces increased production of many metabolites initially produced in low quantities by *F. verticillioides*, particularly fusarins. This visualization highlights the ability of EMs to alter gene expression and, in the case of SV, to inhibit DNA deacetylation in fungal strains. SV significantly increased the production of 39 out of the 50 most discriminant metabolites compared to the control condition. We can see that the addition of SB and NIC has little impact compared to the control condition on the 50 most discriminant metabolites between conditions.

**Figure 8:**
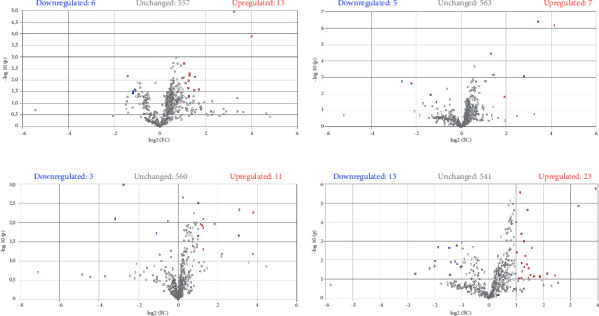
Volcano plot representing the response of *F. verticillioides* to treatment with the different epigenetic modifiers: AZA (a), SB (b), NIC (c), and SV (d) compared with the control condition (PDB + FV). Each point represents a variable (m/z_Rt). Blue and red colors represent significant changes (*p* value <0.05 and fold-change ratio > 2) in the distribution of variables in the presence of epigenetic modifiers. Upregulated metabolites are shown in red, downregulated metabolites in blue, and unchanged metabolites in gray.

To more precisely observe the influence of EMs on the production of metabolites compared to the control condition, we conducted a pairwise comparison. This comparison was carried out in the form of a volcano plot (Figure 8), comparing each condition with the addition of an EM against the control condition (*p* value < 0.05 and fold-change ratio > 2). This visualization enables the identification of metabolites that are significantly up- or downregulated between the two experimental conditions. Among all metabolites, 13, 7, 11, and 23 metabolites were overexpressed when *F. verticillioides* was cultured in the presence of AZA (Figure 8(a)), SB (Figure 8(b)), NIC (Figure 8(c)), and SV (Figure 8(d)), respectively. In contrast, 6, 5, 3, and 13 metabolites were underexpressed in the presence of AZA, SB, NIC, and SV, respectively. Last, the production of 557, 563, 560, and 541 metabolites was not significantly impacted by the presence of AZA, SB, NIC, and SV, respectively. Among the metabolites significantly underexpressed by the addition of AZA, we found fusarin A and one of its analogs, as well as dihydrofusarin C. No fusarin was upregulated in this condition. SB, NIC, and SV did not impact the production of fusarins as they did not significantly affect the production of these molecules compared to the control condition. We saw in the heat map that SV significantly increased a cluster of molecules including fusarins when compared to other conditions. However, when only comparing with the control condition, this effect is not significant. SV is the EM that had the most effect on the metabolome of *F. verticillioides* as it significantly increased the production of the largest number of metabolites. The second most effective modifier under the applied conditions on *F. verticillioides* is AZA, followed by NIC. SB is the EM that worked the least for inducing the production of specialized metabolites by *F. verticillioides*.

The various results obtained suggest that SV is the EM with the greatest effect on the metabolome of *F. verticillioides*. Its efficacy can be explained by its action as a class I HDAC inhibitor, inducing hyperacetylation of histone H4 and promoting the activation of several transcription promoters. SV might also affect the DNA methylation status in several types of cells [45]. The second EM impacting the alteration of the metabolomics profile is AZA, a cytidine derivative. In contrast, SB and NIC, sharing the same mechanism of action as SV, did not show the same efficacy in modulating the metabolome of *F. verticillioides*. The two most effective EMs, SV and AZA, are linked to DNA methylation inhibition. It would then be interesting to compare these results with another EM that modulates the state of DNA methylation and to test different concentrations of EMs, as those used in this study come from the literature [34, 36]. In addition, cytotoxicity tests, such as WST-1, could be valuable for future studies to verify the effects of EMs on fungal growth and metabolite production.

## 4. Conclusions

This study investigated the impact of EMs on the metabolome of *F. verticillioides*, with a particular focus on mycotoxin production. Our findings highlighted the significant influence of HDAC inhibitors and DNA methylation inhibitors on the fungal metabolic profile, notably on fusarin production, that are of interest since they might have mutagenic and carcinogenic activities [47, 48]. This research underscores the potential of EMs to reprogram gene expression and alter secondary metabolite production in *F. verticillioides*.

We chose to work with PDB due to its versatility in supporting the growth of a wide range of fungi, its liquid nature facilitating the addition of EMs, and its common use in mycological studies. However, our study has certain limitations, including the exclusive use of PDB medium, which is not optimal for all mycotoxin production, and the absence of transcriptomic and global methylation analyses. To deepen our understanding, future research should incorporate transcriptomic and global methylation analyses, utilize alternative culture media, such as maize flour agar, optimize compound extraction methods to identify a broader range of metabolites, and confirm the chemical structure and cytotoxic activity of identified molecules.

In the context of climate change, which favors fungal contaminations, this research opens interesting perspectives on the use of EMs to study the mycotoxigenic potential of fungi. Nevertheless, it also highlights the complexity of genetic regulation mechanisms, reminding us that predicting metabolite production from fungal DNA remains a challenge.

## Figures and Tables

**Table 1:** Summary table of the annotations of metabolites present in the crude extracts of *F. verticillioides* cultured in PDB alone or treated with AZA, SB, NIC, or SV, as well as the level of annotation, the m/z, the Rt, and their distribution in the different conditions.

m/z	Rt (min)	Annotation	Annotation level	Occurrence
180.1011	4.852	Fusaric acid	2.4 MS-FINDER	AZA = SB = NIC = SV = PDB
383.0764	8.895	Bikaverin	2.1 MS-FINDER	SV > PDB > SB > NIC > AZA
384.1803	7.823	Fusarin A analog	GNPS 0.86	SV > PDB > NIC > SB > AZA
384.1807	8.03	Fusarin A analog	GNPS 0.77	SV > PDB > SB > NIC > AZA
398.1961	8.564	Fusarin A analog	GNPS 0.79	SV > PDB > NIC > SB > AZA
400.2115	7.652	Fusarin A analog	GNPS 0.71	SV > SB > PDB > NIC > AZA
402.2273	8.142	Fusarin A analog	GNPS 0.84	SV > PDB > NIC > SB > AZA
402.2274	8.005	Fusarin A analog	GNPS 0.82	SV > NIC > PDB > SB > AZA
414.1913	7.833	Fusarin A analog	GNPS 0.79	SV > NIC > PDB > SB > AZA
416.2066	7.694	Fusarin A	GNPS 0.92	SV > PDB > NIC > SB > AZA
416.207	8.431	Fusarin A	GNPS 0.8	SV > PDB > NIC > SB > AZA
416.2076	8.708	Fusarin A	GNPS 0.72	SV > PDB > NIC > SB > AZA
432.2019	7.844	Open-chain fusarin C	GNPS 0.87	SV > PDB > NIC > SB > AZA
432.2019	7.683	Open-chain fusarin C	GNPS 0.82	SV > PDB > NIC > SB > AZA
432.202	8.07	Open-chain fusarin C	GNPS 0.83	SV > PDB > NIC > SB > AZA
434.2167	7.184	Dihydrofusarin C	GNPS 0.79	SV > NIC > PDB > SB > AZA
438.1883	8.556	Dihydrofusarin C analog [M+Na]^+^	GNPS 0.69	SV > NIC > PDB > SB > AZA
438.1887	8.707	Fusarin A [M+Na]^+^	Not annotated	SV > NIC > PDB > SB > AZA
438.1889	7.928	Open-chain fusarin C analog or dihydrofusarin C [M+Na]^+^	GNPS 0.68	SV > NIC > PDB > SB > AZA
454.1822	7.033	Fusarin C [M+Na]^+^	GNPS 0.66	SV > NIC > PDB > SB > AZA
454.1825	7.846	Fusarin D [M+Na]^+^	GNPS 0.83	SV > NIC > SB > PDB > AZA
454.1833	7.692	Fusarin C [M+Na]^+^	GNPS 0.84	SV > SB > PDB > NIC > AZA
454.1841	8.035	Fusarin D [M+Na]^+^	GNPS 0.77	SV > SB > NIC > PDB > AZA
456.1984	7.282	Dihydrofusarin C [M+Na]^+^	GNPS 0.72	SV > NIC > PDB > SB > AZA

*Note:* GNPS score represents spectral similarity score between the detected ions and the molecules contained in MetGem databases.

## Data Availability

The data used in this study are available from the author on request.
